# Uncomplicated urinary tract infections in Swedish primary care; etiology, resistance and treatment

**DOI:** 10.1186/s12879-019-3785-x

**Published:** 2019-02-13

**Authors:** Helena Kornfält Isberg, Eva Melander, Katarina Hedin, Sigvard Mölstad, Anders Beckman

**Affiliations:** 1 Department of Clinical Sciences, Malmö, Family Medicine, Lund University, Malmö, Sweden; 2 Regional Centre for Communicable Disease Control, Malmö, Sweden; 3 Futurum, Region Jönköping County and Department of Medical and Health Sciences, Linköping University, Linköping, Sweden; 4 Department of Translational Medicine, Lund University, Malmö, Sweden

**Keywords:** Urinary tract infection, Primary health care, General practice, Antibiotic, Antibiotic resistance, Urine culture, E.Coli

## Abstract

**Background:**

Uncomplicated urinary tract infection (uUTI) is common and a majority of patients are prescribed antibiotics. There is little knowledge about antibiotic resistance in urine samples from patients with uUTI in primary health care (PHC).

The aim was to describe antibiotic treatment, bacterial findings, the prevalence of resistant *E.coli* and factors associated with antibiotic resistance. The aim was also to compare the prevalence of resistant *E.coli* in clinical practice with the prevalence of resistant *E.coli* in routine microbiological laboratory data.

**Methods:**

This observational study in PHC setting started in November 2014 and ended in March 2016. Women aged 17 years and older with symptoms indicating uUTI from eight PHCs were included. Questionnaires were used to retrieve anamnestic data. All urine samples were sent to the local laboratory of microbiology for diagnostic analysis and susceptibility testing. Proportions of resistant *E.coli* were compared with corresponding data from the regional laboratory.

**Results:**

Urine cultures were analysed in 304 women with a median age of 46 (IQR 32–66) years. Bacterial growth was found in 243 (80%) of urine samples, and *E.coli* in 72% of the positive samples. A total of 80% of detected *E.coli* isolates were susceptible to all tested antimicrobials and resistance rates to ciprofloxacin were lower than reported from the local clinical laboratory. Antibiotic treatment within the last year was independently associated with antibiotic resistant *E.coli* in the urine sample adjusted OR 4.97 (95% CI 2.04–12.06). A total of 74% of the women were treated with antibiotics. The most prescribed was pivmecillinam followed by nitrofurantoin.

**Conclusions:**

Antibiotic resistance in *E.coli* was low. Antibiotic treatment within the last year was associated with antibiotic resistant *E.coli*. Data from the clinical microbiology laboratory indicates that laboratory data may overestimate antibiotic resistance and lead to an unnecessary change in empiric antibiotic treatment of uUTI in primary care. The empirically prescribed antibiotics, were inline with Swedish treatment recommendations for uUTI.

## Background

In primary health care (PHC) uncomplicated urinary tract infection (uUTI) is one of the most common reasons for prescribing of antimicrobials to women who are otherwise healthy. Previous data from Swedish PHC show that among women consulting with suspected uUTI, a total of 59–86% were prescribed antibiotics [1, 2].

In general, resistance to clinically important antimicrobials is low in Sweden. However, there are increasing trends of resistance, especially in *Escherichia coli (E.coli*) the most common pathogen causing uUTI. Swedish national data on antibiotic resistance, which is gathered in the national register Res-Net, is based on a systematic report of *E.coli* in urine cultures from the 25 microbiological laboratories in Sweden. The urinary cultures originate from both hospitalized and primary care patients. Resistance to trimethoprim in *E.coli* from these routine laboratory urinary samples has increased from 17.5% in 2007 to 22% in 2016 and resistance to ciprofloxacin has increased from 7.5% in 2012 to almost 10% in 2016. The frequency of strains resistant to cefadroxil has increased from 3.5% in 2007 to 5% in 2016 and about two-thirds of these cefadroxil-resistant strains are ESBL-producing. The resistance rates for mecillinam and nitrofurantoin have been continuously low and were 5 and 1% respectively in 2016 [3].

uUTI is one of the most common infections in primary care. Recent studies from PHC in different European countries have been performed but since the health care systems, antibiotic treatment recommendations and patient populations differ between countries, the studies are not representative for the Swedish PHC population [4, 5]. Previous studies have shown that factors such as high number of antibiotics courses over the previous year, foreign travel, nursing home and hospital residency, increase the odds for antibiotic resistance [5, 6]. There are few Swedish studies describing bacteriological etiology, antibiotic resistance and antibiotic treatment in this group of patients. The most recently published studies, which were performed in 2007 and 2008, with data on antibiotic resistance in patients from Swedish PHC, showed low levels of antibiotic resistance in *E.coli*. There is little knowledge of the factors of relevance for antibiotic resistance in a Swedish PHC population.

Swedish guidelines for the treatment of urinary tract infections suggest pivmecillinam and nitrofurantoin as primary choice antibiotics and prescribers are recommended to minimize the use of fluoroquinolones and trimethoprim because of the resistance situation [7].

Since treatment is initiated empirically, and urine culture is only performed when there are complicating factors, there is little knowledge about the bacterial etiology and resistance situation in PHC. Microbiological data on etiology and bacterial susceptibility presented from laboratories most often represents a selected sample of patients with complicating factors. Bacterial susceptibility varies geographically and with time, thus it is important to monitor patients without complicating factors that account for a high share of antibiotic prescriptions in PHC.

We aimed to describe antibiotic treatment, bacterial findings, the prevalence of resistant *E.coli* and factors associated with antibiotic resistance in patients 17 years and older attending PHC with symptoms indicating urinary tract infection. Our aim was also to compare the prevalence of resistant *E.coli* with the prevalence of resistant *E.coli* in routine microbiological laboratory data.

## Methods

A prospective observational study was performed at primary healthcare centers (PHCCs) in the county of Skåne in the south of Sweden.

### Population

Patients included in the study were women aged 17 years and older attending the PHCC with a suspected uUTI usually due to frequency, urge or dysuria. Excluded patients were those with in-dwelling catheters, signs of pyelonephritis or not capable of understanding and filling out the questionnaire.

### Primary health care center (PHCC)

In 2013 the population of the county of Skåne was 1,272,434 and the total number of PHCCs was 150 [8]. The size of the PHCCs varied from 2 to 3 general practitioners employed to 10–15, and the number and age-span of registered patients and the workload differed between the centers. The number of registered patients at the eight included PHCC varied from 7500 to 14,000 registered patients; the total registered population was 88,670 patients. The selection of included PHCCs was performed strategically without any former knowledge of antibiotic prescription pattern or prevalence of uUTI. The included PHCCs represented both rural (four PHCCs) and urban (four PHCCs) areas of the region, including the city of Malmö with approximately 300,000 inhabitants. Based on previous studies on uUTI in primary care, we estimated that 60% of urine samples would contain bacteria and 80% of positive cultures would be *E.coli* [9]*.* We aimed to collect 400 urine samples to find approximately 200 *E.coli* isolates, which we estimated would be enough to describe the prevalence of resistant *E.coli*.

### Data collection and clinical assessments

Recruitment of patients started on November 1st 2014 and continued until the end of March 2016. Clinicians were asked to recruit all eligible women, 17 years and older with a suspected uUTI.

Patients were given written information about the study and written informed consent was obtained. Mid-stream urine samples were collected. Clinicians were asked to manage the patients according to their usual practice. Patients completed a questionnaire regarding severity of UTI symptoms, previous UTIs, and previous antibiotic treatment before leaving the PHCC. Patients were also asked whether they had travelled abroad (outside of Sweden) and to what countries within the last six months. Bladder incubation time was registered by the patients and they were asked to register if prescription of antibiotics was done. If information concerning antibiotic treatment was unclear or not complete, the researcher examined the patient electronic medical record to retrieve complete information.

### Urine culture

Specimens were stored at + 4 degrees Celsius until transportation to the laboratory on the day of collection, using routine transport systems. Samples that were not sent to the laboratory within 24 h from collection were destroyed. The urine samples were sent to the Department of Laboratory Medicine, Division of Clinical Microbiology in Lund for microbiological analysis.

Identification to the species level was performed by using approved conventional methods [10]. Growth of bacteria was considered significant if the number of colony-forming units (CFU)/mL was ≥10^3^ for primary pathogens as *E. coli* and *S. saprophyticus* and ≥ 10^4^ CFU/ml for secondary pathogens [11].

Antimicrobial susceptibility testing followed guidelines and breakpoints proposed by the European Committee on Antimicrobial Susceptibility Testing (EUCAST) [12]. *E.coli, Klebsiella pneumoniae* or *Proteus mirabilis* resistant to cefotaxime and/or ceftazidime were confirmed as ESBL-producing bacteria by the MAST test (MAST, UK) [13]. Strains with reduced susceptibility to carbapenems were examined with Check-MDR (http://www.check-points.eu).

The proportions of resistant *E.coli* isolates in the study were compared to the proportion of resistant *E.coli* isolates from the regional laboratory of microbiology in Lund including cultures from both PHC and hospital care for the same time period and the same age groups.

Patients were only included in the study once and only one isolate per individual was included in the regional laboratory data.

### Statistical analysis

This was a prospective observational study*.* All patient data were anonymized before analyses were initiated*.* Questionnaire and laboratory data were collected using Microsoft Excel and statistical analysis was performed using SPSS 22.0 software (IBM, Armonk, NY, USA). Descriptive statistics (median, IQR, mean + SD, percentages) were used to describe bacterial findings, antibiotic resistance in *E.coli* and antibiotic prescriptions. Comparison between proportions for categorical variables in two independent groups was performed using the two-sided χ^2^ test. A *p*-value < 0.05 was considered statistically significant. Binary logistic regression was used to model the correlation between antibiotic resistance in *E. coli* and several independent variables. Odds ratios (OR) with 95% CI were calculated*.*

### Ethics

The study was approved by the Regional Ethical Review Board in Lund, Sweden, (Dnr 2014/432).

## Results

A total of 324 women with UTI symptoms agreed to participate in the study. Urine cultures were analyzed in 304 women. Only women with analyzed urine cultures were included in the final presentation of data.

### Patient characteristics

The 304 included women had a median age of 46 (IQR = 32–66) years. A total of 121 patients reported being treated with antibiotics due to UTI once or more during the last 12 months, the median number of antibiotic courses due to UTI reported was 1 (IQR = 1–3).

Diabetes was reported in 3.6% and incontinence in 18.5% of women. Almost half of the patients had experienced a uUTI more than five times prior to the study visit and half of the patients had experienced a uUTI episode during the last six months.

The median number of months since last UTI was seven months (IQR 1.1–24.0). The median number of days of symptom duration at visit was four (IQR 2–7) and median bladder incubation time was 2.5 h (IQR 1.2–4.0). A total of 55 (17%) patients reported having been abroad within the last six months.

### Bacterial growth in urine samples from included patients

Bacterial growth was found in 243 (80%) of urine samples. *E.coli* and *S. saprophyticus* were the most common pathogens (Table 1). In 20 cultures (8%) the laboratory reported growth of two relevant species. When two species were found only the most relevant pathogen, which was *E.coli* in 18 and *Klebsiella pneumoniae* in two has been considered in this study.

**Table 1:** Distribution of bacterial species in urine cultures in women with symptoms suggestive of uncomplicated UTI

Bacterial species	No of samples with bacterial growth n (%) (*n* = 243)
*Escherichia coli*	176 (72)
*Staphylococcus saprophyticus*	22 (9)
Other Gram-pos spp^a^	18 (7)
Other Gram-neg spp^b^	13 (5)
*Streptococcus group B*	10 (4)
Other^c^	4 (2)

^a^Other Gram-positive spp.*: 6 Enterococcus spp, 1 S****.*** *aureus, 2 Coagulase-negative staphylococci* other than *S.saprophyticus*, *2 Streptococus group G, 5 Gram-positive bacteria mixed growth, 2 Lactobacillus, 1 Aerococcus urinae*

^b^Other Gram-negative spp.: 5 *Klebsiella* spp., 3 *Citrobacter*, 2 *Enterobacte*r spp., 1 *Proteus* spp., 1 *Pseudomonas* spp., 1 *Haemophilus influenzae*

^c^Other: 1 *Gardnerella vaginalis, 3 mixed growth of bacteria*

### Antibiotic resistance in *E.coli* isolates-comparison with data on antibiotic resistance from the local clinical microbiology laboratory

The overall antibiotic resistance for *E. coli* is presented in Table 2. In total, 80% of detected *E.coli* isolates were susceptible to all tested antimicrobials. No resistance for nitrofurantoin was found.

**Table 2:** Comparison of antibiotic resistance for *Escherichia coli* in the study and in routine laboratory data^a^

Antibiotic tested/resistance mechanism	Resistance in primary care No of samples n (%) (*n* = 176)	Resistance reported from the laboratory^b^ No of samples n (%) (*n* = 23,179)	*p-*value
Mecillinam	2 (1.1)	714 (3.1)	0.18^c^
Nitrofurantoin	0 (0)	184 (0.8)	0.66^c^
Trimethoprim	30 (17.0)	4612 (19.9)	0.35^d^
Cefadroxil	4 (2.3)	1223 (5.3)	0.087^c^
Ciprofloxacin	2 (1.1)	1400 (6.0)	0.0022^c^
ESBL-producing	4 (2.3)	807 (3.5)	0.53^c^

^a^Urine samples from women during November 2014 to March 2016

^b^The Department of laboratory medicine, Division of Clinical Microbiology in Lund

^c^The Fisher exact test

^d^Chi^2^ test

Combined resistance was rare. Four *E.coli* isolates were resistant to trimethoprim and one or two more antibiotics (mecillinam (1), cefadroxil (2) cefadroxil and ciprofloxacin (1)). ESBL was identified in four isolates, two of these were trimethoprim resistant and one ciprofloxacin resistant.

Difference in prevalence of antibiotic resistance in *E.coli* between urine samples in the present study and the local clinical laboratory of microbiology was found for ciprofloxacin (*p* = 0.0022) (Table 2).

### Factors of importance for antibiotic resistance

Treatment with UTI antibiotics within the last 12 months was associated with increased odds for antibiotic resistance in *E.coli* (adjusted OR 4.97 95% CI 2.04–12.06). No association between the number of prescriptions of UTI antibiotics within the last year and resistant *E.coli* was found (adjusted OR 1.35 95% CI 0.97–1.87). Travelling abroad within the last six months increased the odds for resistant *E.coli* (adjusted OR = 4.02 95% CI 1.35–11.35). There was no significant association between antibiotic resistance and age, tested both by year and age groups, incontinence and diabetes (Table 3).

**Table 3:** Risk factors for carriage of resistant *Escherichia coli* ^a^ among women with UTI symptoms (*n* = 176)

Variable	Crude OR (95% CI)	Adjusted^b^ OR (95% CI)
Age (per year)	1.00 (0.98–1.02)	0.99 (0.97–1.01)
Incontinence	1.00 (0.99–1.02)	0.83 (0.39–1.80)
Diabetes	7.60 (0.67–86.26)	2.32 (0.13–39.77)
Abroad last 6 months	1.64 (0.66–4.10)	4.02 (1.35–11.95)
Treatment with UTI antibiotics within the last 12 months^c^	3.83 (1.72–8.54)	4.97 (2.04–12.06)

^a^Growth of *E.coli* resistant to either mecillinam, trimethoprim, ciprofloxacin, cefadroxil or ESBL producing

^b^Method: Log regression, enter, adjusted for; age, incontinence, diabetes, abroad last 6 months and treatment with UTI antibiotics within the last 12 months

^c^Antibiotic treatment reported by the patients

### Antibiotic prescriptions

In total 74% of the women were prescribed empirical antibiotics (i.e. antibiotics prescribed at consultation without information about results from urine culture). Presented data are prescriptions done at consultation. Pivmecillinam was the most commonly prescribed antibiotic, followed by nitrofurantoin. Trimethoprim and fluoroquinolones were prescribed to a low extent (Table 4).

**Table 4:** Empiric antibiotic treatment of uncomplicated urinary tract infection

Treatment *n* = 324	n (%)
No antibiotic	78 (24.1)
Any antibiotic	239 (73.8)
Pivmecillinam	132 (55.2)
Nitrofurantoin	92 (38.5)
Trimethoprim	5 (2.1)
Ciprofloxacin	5 (2.1)
Other^a^	5 (2.1)
Data not available	7 (2.1)

^a^Cefadroxil, Trimethoprim/sulfamethoxazole, Doxycycline

Appropriate antibiotics were prescribed to 73% of the patients (patients with growth of bacteria sensitive to the prescribed antibiotic and patients with no growth of bacteria and no antibiotic prescribed). 63% of women had a UTI with bacteria sensitive to the prescribed antibiotic and 10% did not have a UTI according to urine culture and were not prescribed antibiotics. Antibiotics were prescribed non-concordantly to 49% of patients with a culture negative for UTI and to 3% of patients with bacteria resistant to the prescribed antibiotic, 14% of patients with growth of bacteria in urine samples were not prescribed antibiotics.

## Discussion

### Main findings

This observational study including women with suspected uUTI in primary care showed bacterial growth in 80% of urine samples. Resistance levels in *E.coli* were low, except for trimethoprim. The resistance levels for ciprofloxacin in *E.coli* were significantly lower than in routine laboratory data. Almost 74% of the women were treated with antibiotics of which the majority were primary choice antibiotics according to clinical guidelines.

### Strengths and weaknesses

This is one of few studies describing bacterial findings in patients with suspected uUTI in primary care. The study design was aimed to be as similar to routine clinical practice as possible. Patients, instead of GPs, were asked to register prescribed antibiotics in the questionnaire, in order to interfere with the GPs’ prescribing as little as possible. In daily clinical practice, urine cultures from adult female patients with UTI symptoms are not analyzed and subsequently there is little knowledge about the prevalence of antimicrobial resistance among women with uUTI in primary health care. A strength of our study is that all urine cultures were analyzed at the same laboratory and that we were able to collect routine data (representing both PHC and hospital care since the laboratory does not separate primary care cultures from hospital care cultures when reporting data on resistance) on resistance in *E.coli* for the same time-period and the same agegroups from that laboratory. We had a high response rate regarding the questionnaires and a low internal drop out. This means that the non -response bias is low and our results are representative for the population included in this study.

It is a weakness of our study that we did not recruit the stipulated 400 patients. Since uUTIs often are managed fast in practice, we believe that patients and GPs did not take the extra time needed to fill out the questionnaire and to inform patients about the study. The total number of patients diagnosed with uUTI at included PHCCs during the study period is not known and thus the number of patients that declined to participate cannot be presented. Bladder incubation time was shorter than the recommended four hours [7]. Due to UTI-symptoms, patients often have difficulties to keep the urine in the bladder for the requested four hours. Antibiotic prescriptions followed recommendations to a high extent. It is possible that GPs, who were aware that their patients were participating in a study, prescribed antibiotics more prudently and were following guidelines to a higher extent. This issue has been described in former studies where GPs who actively chose to take part in a medical audit or who were participating in research networks prescribed fewer antibiotics than the total population of GPs [14, 15].

### Previous studies

In this current study, bacterial growth was found in 80% of urine samples and growth of *E.coli* was identified in 72% of positive cultures. This is inline with other studies that report that *E.coli* causes the majority of infections among outpatients regardless of age group [16, 17]. In a previous study from out-patients in Sweden, *E.coli* was found in 77% of positive urine cultures from women [9].

The proportions of resistant *E.coli* isolates in our study were compared to the proportion of resistant *E.coli* isolates from the regional laboratory of microbiology during the same time period. Our data, representing women with suspected uUTI attending PHCCs, shows lower prevalence of resistance in *E.coli* to ciprofloxacin, which could be expected given that information on antibiotic resistance is often based on selected laboratory data with more severely ill patients. Thus in order to correctly describe antibiotic resistance in *E.coli* from PHC we need laboratories to be able to separate information about antibiotic resistance in isolates from PHC from isolates from hospital care.

Compared to three previous primary health care studies from different European countries our data on antibiotic resistance in *E.coli* shows lower resistance rates to ciprofloxacin but similar rates to trimethoprim, nitrofurantoin and cefadroxil [4, 5, 18]. In a PHC study in our neighboring country Denmark resistance rates to trimethoprim and ciprofloxacin in *E.coli* have been reported to 23 and 8% [19]. Also ESBL producing *E.coli* was more common in uUTI in Denmark as compared to Sweden [19]. Previous studies have shown large variations in antibiotic prescribing for uUTI in various countries and this may be one reason among several behind the difference in antibiotic resistance between countries [18].

Our results show that treatment with UTI-antibiotics within the last 12 months was associated with antibiotic resistance in *E.coli (p < 0.001)*, which is in line with previous UTI studies where antibiotic resistance is associated with previous antibiotic treatment, i.e. with the strongest effect shown in the month directly after prescription but detectable up to 12 months [20]. Antibiotic consumption within the previous four weeks did not significantly affect the prevalence of resistant *E.coli* in our analysis and since it could interact with the variable antibiotic consumption during the last year, we excluded it in the regression analysis. By contrast to other studies, we could not find that the number of prescriptions of UTI antibiotics within the last year increased the odds for resistant *E.coli* [5, 21, 22]. This may be due to a rather small group of resistant *E.coli* in our material, other antibiotic choices and shorter duration of treatment than in previous studies. Only a few patients with growth of *E.coli* (*n* = 25) reported being treated with antibiotics within the last four weeks, due to the low number the result has to be interpreted with caution. Travelling abroad has in previous studies showed associations with ESBL in UTI [6]. In our study, travelling abroad within the last six months was associated with resistant *E.coli* (adjusted OR 4.02%CI 1.35–11.95).

In 2007 recommendations for the treatment of uUTI in women were published. Pivmecillinam and nitrofurantoin are recommended as first choice antibiotics, the use of cephalosporins or fluoroquinolones is no longer recommended in Sweden when treating uUTI [7]. In a Swedish PHC study on uUTI performed in 2008, one year after the publication of the recommendations, 59% of women were treated with antibiotics, trimethoprim was prescribed to 17% and fluoroquinolones to 6% [2]. This is in contrast to the present study where 74% of the women were treated with antibiotics. Trimethoprim and fluoroquinolones were prescribed to 2% of women respectively, in line with treatment recommendations. The difference in prescribing between the two studies reflects that one year is not enough to alter the prescribing pattern; it takes time before new guidelines are implemented in clinical practice.

The proportion of patients prescribed UTI antibiotics differs markedly between different European countries and range between 56 and 99% in previous European studies [23]. The wide range of antibiotic prescribing is probably due to uncertainty in diagnostic testing [23] but also likely due to differences in healthcare organization, accessibility to GPs and adherence to treatment recommendations. Previous studies suggest that strategies such as a follow up consultation or delayed prescriptions of antibiotics could decrease the high rate of antibiotic prescriptions in uUTI in primary care [24, 25]. The uncertainty in diagnostic testing may lead to both under- and overprescribing of antibiotics. In the present study 14% of patients with growth of bacteria in urine samples were not prescribed antibiotics and 49% of patients with no significant growth in urine samples were treated with antibiotics. Previous studies suggest that empirical treatment may result in up to 40–60% of women with UTI symptoms receiving antibiotics in spite of negative culture [26, 27]. It is required that better diagnostic tools are developed to target antibiotic prescription and that selected patients are offered delayed prescriptions in order to avoid unnecessary prescribing and reduce antibiotic use.

### Relevance

In 2007, Swedish guidelines for the treatment of uUTI in women were published. As a result of increasing resistance levels to trimethoprim and ciprofloxacin in *E.coli,* pivmecillinam and nitrofurantoin were recommended as first choice antibiotics when treating uUTI [7]. Based on the results on resistance levels from the present study, pivmecillinam and nitrofurantoin are good first choice forms of treatment. The included, patients were prescribed antibiotics according to guidelines. Even though the use of pivmecillinam and nitrofurantoin was high, resistance levels were low and we found no *E.coli* strain resistant to nitrofurantoin.

The study also shows that resistance rates to trimethoprim are high in PHC, accordingly trimethoprim should only be used after urine culture. Further, resistance rates to ciprofloxacin were lower among patients included in this study compared with data from the clinical microbiology laboratory. The use of fluoroquinolones (ciprofloxacin) should still be avoided in the treatment of uUTI since the use of fluoroquinolones has been associated with the rise in prevalence of multi resistant bacteria. Prescribing should be limited to complicated cases and preferably after culture results.

## Conclusion

A majority of patients were treated with antibiotics according to clinical recommendations. Antibiotic resistance in *E.coli* in urine samples from patients with suspected uncomplicated UTI in primary health care was low except for trimethoprim, confirming that pivmecillinam and nitrofurantoin are good first options.  The level of resistance to ciprofloxacin was lower than routine data from the clinical microbiology laboratory indicating that laboratory data overestimates antibiotic resistance and may lead to an unnecessary change in empiric antibiotic treatment of uUTI in primary care. To better address resistance levels among patients in PHC, reports on resistance data from the local clinical laboratory have to separate information about antibiotic resistance in isolates from PHC from isolates in hospital care.

## Abbreviations

CFU: Colony forming units
E. coli: *Escherichia coli*
ESBL: Extended spectrum beta lactamase
GP: General practitioner
PHC: Primary Health Care
PHCC: Primary Health Care Center
S. saprophyticus: *Staphylococcus saprophyticus*
UTI: Urinary tract infection
uUTI: Uncomplicated urinary tract infection
